# A case of fever and unexplained acute renal failure

**DOI:** 10.4103/0971-4065.42353

**Published:** 2008-04

**Authors:** V. Sakhuja, R. Agarwal, N. Kalra

**Affiliations:** **CPC Editor :** Additional Professor of Histopathology, PGIMER, Chandigarh, India; **CPC Chairperson :** Dean and Head of Nephrology, PGIMER, Chandigarh, India; **Clinical Discussant :** Assistant Professor of Pulmonary Medicine, PGIMER, Chandigarh, India; **Radiology Discussant :** Assistant Professor of Radiodiagnosis, PGIMER, Chandigarh, India

A 45-year-old male was admitted with history of fever for 3 weeks and abdominal pain and dysuria for the last 20 days, generalized swelling of the body for 15 days, and increasing breathlessness and decreasing urine output for the last 4 days. No h/o hematuria, pyuria, graveluria; h/o nausea, vomiting, altered sensorium;and h/o orthopnea, paroxysmal nocturnal dyspnea or seizures.

**Past history:** Hypertensive - 11 years, pulmonary tuberculosis since 1992, received ATT for 3 years. Smoker (two pack beedies/day × 25 years) and h/o cough with mucoid expectoration since 1995.

**On examination:** Patient conscious, PR - 92/min, regular; BP - 140/90 mmHg; RR - 30/min. There was pallor and pedal edema. No icterus, cyanosis, clubbing or lymphadenopathy. JVP was normal. Abdomen - No organomegaly, flank and renal angle tenderness + Nervous system - Flaps +, No meningeal signs or focal neurologic deficit; Respiratory system - Barrel chest, expiratory polyphonic wheeze; CVS - Normal.

**Table:** Investigations

	9/8/07	13/8/07	16/8/07	18/8/07	22/8/07	25/8/07	28/8/07	5/9/07	7/9/07	9/9/07
Biochemical investigations										
Na/K (mEq/l)	138/7.2	135/5.6	137/4.4	142/4.5	139/3.4	136/3	135/3.3	137/3.9	139/4.9	128/6.4
BU/Creat (mg%)	160/14.4	116/11.4	104/9.8	65/7	136/8.6	135/8	118/6.8	117/7.4		
Bilirubin (mg%)		0.6	0.5	0.6	0.5	0.6	1.1	0.7		
Prot/Alb (gm%)			5.9/2.1	6.4/2.3	6.3/2.4	6.2/2.5		6.6/1.9	6.6/2.4	
AST/ALT			29/20	28/21	33/24	20/18				
SAP			394	379	267	225	352	841	727	
Ca/PO_4_			7.5/5.3	8/8.9		8.1/8.8		9.2/4.7	8/8.9	
RBS		102						60		
Arterial blood gases										
PH	7.37	7.35	7.42	7.38					7.43	7.3
PaO_2_	100	55	67	56					50	62
PaCO_2_	36	33	27	39					32	30
HCO_3_	21	18	17	20					21	14
SaO_2_	97	88	94	89					87	90
FiO_2_	NA	8LPM	0.21	0.21					12LPM	0.2
Coagulation profile										
PT				16			18			
PTTK				32			38			
Fibrinogen				5.02						
D-dimers				+ve						

**Table:** Urinalysis

	16/8/7	23/8/07	5/9/07
RE	Albumin - nil; sugar - nil		Albumin - 4+; sugar - nil
ME	N	20-25 pus cells	80-100 RBCs;
			25-30 pus cells

**Table:** Ascitic fluid

	19/8/07	25/8/07	7/9/07
TLC	600	240	130
DLC	80/20		
Protein/sugar	2.7/180	2.1/109	1.8/39
SAAG	1.6		
ADA	14		
AFB	Negative		
Cultures	Sterile	*E. coli*	

**Table:** Complete blood count

	9/8/07	16/8/07	22/8/07	28/8/07	5/9/07	7/9/07	9/9/07
Hb (g/dl)	12.1	8	7.9	6.4	6.9	7	9.7
TLC	35000	24700	31900	35600	19100	18700	4000
DLC		72/23/4/1	92/2/1/1	82/14/3/1	64/26/2/2/6	57/38/3/2	90/8/1/1
			My_2_/Mmy_2_				
Platelets (/mm^3^)		2.28 lacs		2.54 lacs	69000	91000	
ESR (dmm/h)		65					
PBF		Mild aniso micro, mild hypo				Mild aniso micro, mild hypo	N/N

EKG - sinus tachycardia, HBsAg - positive, Anti-HCV - negative, HIV - non-reactive, abdominal fat pad aspiration - negative for amyloid, Urine Bence-Jones protein - ×3 negative, serum electrophoresis - normal

**Table:** Microbiology

	16/8/07	18/8/07	20/8/07	23/8/07	24/8/07	30/8/07	2/9/07
Blood cultures		×2 Sterile			×2 Sterile		×2 Sterile
Sputum cultures	*K. pneumoniae*			*Ac. calcoaceticus*	*Ac. calcoaceticus*		
				*C. tropicalis*			
Urine cultures	*Ps. aeruginosa*		Sterile	Sterile			
PCN fluid cultures					Sterile	Sterile	

Sputum AFB (×2) - negative, Urine AFB (×3) - negative, PCN fluid AFB - negative

## Radiology

Serial CXRs - RUL fibrosis, bilateral calcific specks.

**Table:** Ultrasound Findings

9/8/07	RK 12.3 LK 11.6; increased cortical heterogeneity; CMD accentuated; PCS compact; mild ascites.
17/8/07	Hepatomegaly (15.5 cm); Normal outline/echotexture; Hepatic Vein-N; Portal Vein-14 mm, prominent splenoportal axis; mild ascites; RK 13.1 LK 13.2; increases cortical heterogeneity; CMD accentuated; PCS- LPCS 7.5 mm (R) 7.2 mm (L); 5 mm calculus in upper group calyces; mild dilatation of upper ureters; urinary bladder and pelvis moderately distended; internal echoes PCS.
18/8/07	Minimal splitting of PCS even after iv lasix 40 mg.
23/8/07	Minimal splitting of PCS even after iv lasix.
24/8/07	RK 13.1 LK 13.2; increases cortical heterogeneity; CMD accentuated; PCS - LPCS 7.5 mm (R) 7.2 mm (L); prominent upper ureters; urinary bladder empty; internal echoes PCS
29/8/07	Minimal splitting of PCS 3 mm; US guidance pigtail placed; urine draining
31/8/07	RK enlarged with loss of CMD; PCS decompressed; no urine draining
2/9/07	PCN catheter coursing through middle calyx with tip projecting into renal parenchyma; PCS not dilated
7/9/07	PCN catheter in situ; well decompressed PCS

### Dr. Naveen Kalra

**Fig. 1:**
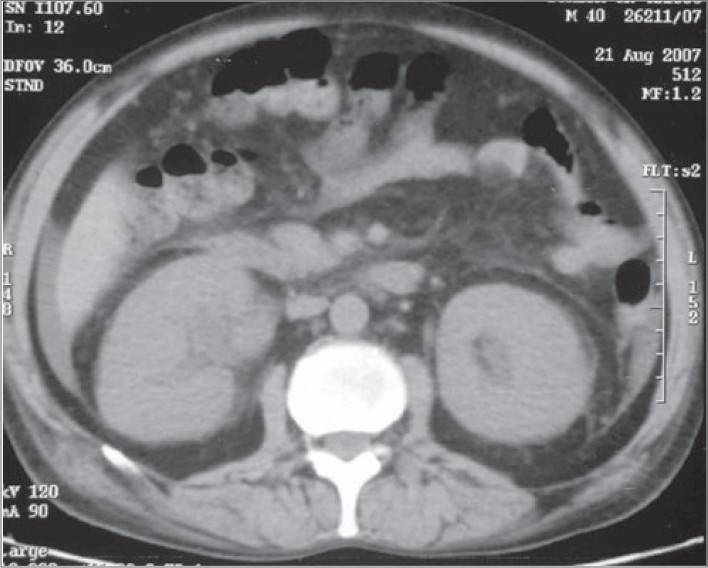
NCCT axial sections at the level of interpolar region of both kidneys show that both the kidneys are enlarged with presence of perinephric stranding. Free fluid is also present in the abdomen

**Fig. 2:**
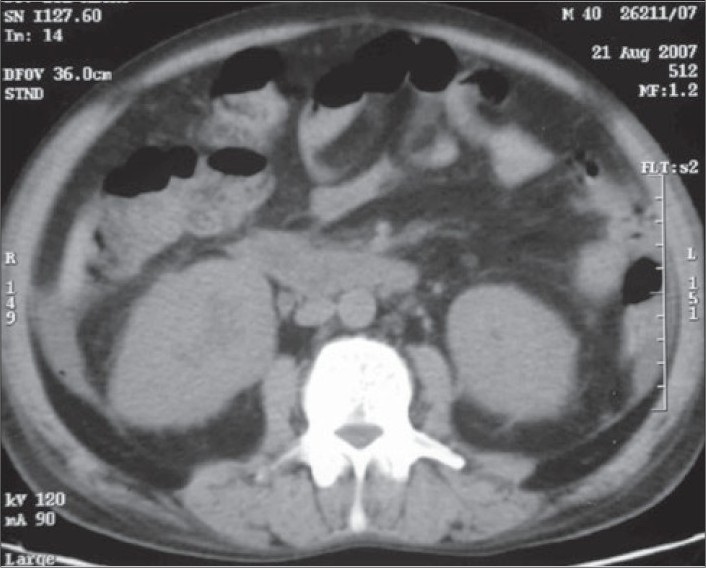
Axial CT sections at the level of the pelvis shows presence of ascites

During the course of the hospital stay, the patient had multiple chest X-rays, ultrasound of the abdomen, and a non-contrast CT (NCCT) of the KUB region. The chest X-rays were essentially normal. The USG showed mild pelvicalyceal dilatation on both sides. The NCCT KUB showed that both the kidneys were enlarged with presence of perinephric stranding [Fig. 1]. Free fluid was also present in the abdomen and pelvis [Fig. 2]. The differential diagnosis of the bilateral nephromegaly would be proliferative/necrotizing disorders, amyloidosis, leukemia/lymphoma, multiple myeloma, and mucormycosis.

## Course and Management

This 45-year-old male admitted with fever, abdominal pain and dysuria followed by generalized edema, increasing dyspnea, and oliguria. Investigations revealed leukocytosis, anemia, azotemia, and hypoalbuminemia. He was found to have enlarged kidneys with doubtful mild hydronephrosis. He was also found to have portal hypertension with high SAAG ascites, and developed SBP during the hospital stay. The patient remained oliguric throughout the hospital stay, received 11 sessions of hemodialysis and IV antibiotics. PCN was performed for suspected hydronephrosis. However, the patient did not improve, and developed hypotension followed by cardiorespiratory arrest from which he could not be revived.

## Unit's Final Diagnosis

- Chronic obstructive pulmonary disease
- Chronic liver disease - HBV-related
- Spontaneous bacterial peritonitis
- Acute pyelonephritis
- Acute renal failure - acute interstitial nephritis/acute tubular necrosis

### Dr. Ritesh Aggarwal

In summary, the patient was a 45-year-old male with history of hypertension and old-treated pulmonary tuberculosis (PTB); a long-time smoker with history suggesting chronic bronchitis. He was also HBsAg positive with findings suggestive of portal hypertension. He had presented with acute urinary tract infection (UTI) with acute renal failure (ARF) with bilaterally enlarged kidneys and doubtful mild hydronephrosis.

The patient did have old-healed PTB. This is supported by the history of intake of anti-tubercular therapy for 3 years and the presence of a fibrotic scar in the right upper lobe. The presence of chronic tobacco smoking with history of chronic bronchitis and diffuse expiratory polyphonic wheeze on physical examination suggests the presence of chronic obstructive pulmonary disease. The hypertension was probably essential. The finding of HBsAg positivity and portal hypertension (as indicated by a high serum ascitic albumin gradient ascites and dilated portal vein on ultrasonography) with occurrence of spontaneous ascitic fluid infection of the monomicrobial non-neutrocytic bacterascites variety suggests the presence of chronic liver disease (chronic viral hepatitis - HBV-related), which got decompensated by the current acute illness. Did these illnesses in any way contribute to the patient's terminal illness? Probably not and are likely to be innocent bystanders.

The patient presented with bilaterally enlarged kidneys and ARF. There is neither a history of diabetes mellitus nor the investigations reveal any hyperglycemia. Similarly, the ultrasound of the kidneys did not reveal any cysts. Hence, these two possibilities - diabetes mellitus and polycystic kidney disease - can be excluded further from the discussion.

Myeloma kidney usually presents with chronic renal insufficiency. However, ARF can be the presenting feature if there is superadded renal insult such as infection or uncontrolled hypertension. However, these patients have significant history of weakness, weight loss, and bone pain, which this index case did not give. Furthermore, the absence of proteinuria at onset, a negative urine examination for Bence-Jones protein, and a normal serum electrophoresis makes this diagnosis unlikely.^1^

Similarly, renal amyloidosis generally presents with chronic renal insufficiency and ARF can occur if there is a superadded renal insult or de novo in a small subset of patients.^2^ The history of old pulmonary tuberculosis also supports the possibility of secondary amyloidosis; however, the absence of proteinuria at onset, the absence of any other system involvement, and a negative abdominal fat pad aspiration again make this diagnosis unlikely.^3^

Tuberculosis is a great mimic; renal tuberculosis, however, presents with scarred kidneys.^4^ Rarely, renal involvement by tuberculosis can manifest as tuberculous interstitial nephritis, which presents with enlarged kidneys. However, most of these patients have active tuberculosis elsewhere, and urine and percutaneous nephrostomy fluid was negative for acid-fast bacilli, which makes this diagnosis unlikely.^4^

Thus, I am left with two possibilities, which are renal mucormycosis and renal lymphoma. Renal mucormycosis classically presents with fever, flank pain, dysuria, pyuria, and lack of response to antibiotics, which was seen in this patient.^5^ However, there are several odd features for this diagnosis. Most patients have significant perinephric collections with multiple hypoechoic lesions on ultrasound examination and multiple low-attenuation areas on computed tomographic scans. Moreover, gross hematuria is common because ARF is usually the result of near total occlusion of the renal arteries.^5^,^6^ A normal urinalysis at the outset, thus, goes against this possibility.

Can the renal disease be related to latent HBV infection? Several types of glomerular lesions have been described in HBV infection, with the most common being membranous glomerulonephritis and membranoproliferative glomerulonephritis.^7^ However, enlarged kidneys are a rare occurrence in these conditions, and normal urinalysis at the onset makes this a very rare possibility.

Renal lymphoma is a classical cause of bilaterally enlarged kidneys and ARF.^8^ Fever, dysuria, and loin pain are usual findings in patients with renal lymphoma and in many instances are mistaken for a UTI.^9^ The presence of mild hypercalcemia and normal urinalysis favors this diagnosis. The mild hydronephrosis is probably secondary to secretory hydronephrosis or ureteric involvement by lymphoma. The urine microbiology seen in this patient is probably secondary to prolonged catheterization rather than a primary event. Finally, the presence of HBV infection is associated with an increased occurrence of lymphoma, and this is another supporting point.^10^

Terminally, the patient had sudden onset hypotension which is probably due to pulmonary thromboembolism. Alternatively, hyperkalemia in this patient could have caused a cardiac arrhythmia which led to the demise of this patient.

### Dr Aggarwal's diagnosis

- Acute renal failure - renal lymphoma
- Chronic liver disease - HBV-related
- Old-healed pulmonary tuberculosis
- Chronic obstructive pulmonary disease
- Essential hypertension

*Dr. Sanjay Jain, Professor of Internal Medicine:* We were not sure what we were dealing with. What was very clear was that this patient had signs of renal inflammation. As you can see on CT scan that there is perinephric stranding, there was persistent leukocytosis and pyuria. Now, the point is what is inciting this renal inflammation, whether this is just bacterial or bacterial plus fungal or this is non-infective inflammation, which is possibly because of lymphoma. Renal papillary necrosis is another possibility. Many patients, who may or may not give history of NSIAD, can have acute onset interstitial nephritis, which is one possibility and should always be excluded.

*Dr. Virendra Singh, Additional Professor of Hepatology:* This patient had portal hypertension, but the findings on ultrasound are not suggestive of cirrhosis. There is no thrombocytopenia, which takes away from the possibility of cirrhosis. This type of presentation can be there in the amyloidosis and many features favor possibility of amyloidosis. The sensitivity of the abdominal fat pad aspirate is only about 60% and the biopsy is a gold standard. I keep the possibility of amyloidosis in the liver as well as kidney in this patient.

*Dr. Sakhuja:* There was a marked elevation of serum alkaline phosphatase, I wonder whether that indicates extensive liver infiltration by some process, by the tumor or by amyloid.

*Dr. Pankaj Malhotra, Associate Professor of Internal Medicine:* Apart from the other possibilities, I think the possibility of intravascular lymphoma can also be kept in this patient. In the last CPC discussion by Dr. A Das, the patient has similar presentation with renal failure and oliguria and that patient had intravascular lymphoma.

*Dr. SK Jindal, Professor and Head of Pulmonary Medicine:* I might like to expand the differential diagnosis a little more. In the first chest X-ray, both the hilar areas are prominent and one can suspect a diagnosis of sarcoidosis, which perhaps is involving other organs like kidney.

*Dr. KS Chugh, Emeritus Professor of Nephrology:* Although amyloid is great mimic, but in his particular case it seems to me very unlikely. A patient presenting with a fast renal failure of just a few days duration and anasarca without having gone through nephrotic stage and with such large kidneys is a very unusual course of amyloid disease. There is no history of nephrotic stage in this patient. I do feel that this could be one of the lymphoma like situations rather than anything else.

### Dr. Ashim Das

A complete autopsy was performed on this 45-year-old male of average build. On opening the abdominal cavity, 1500 ml of straw-colored fluid was noted in the peritoneal cavity. There was no excess of fluid in the pleural cavities or pericardial cavities. The lungs were adherent to the thoracic cavity.

**Fig. 3:**
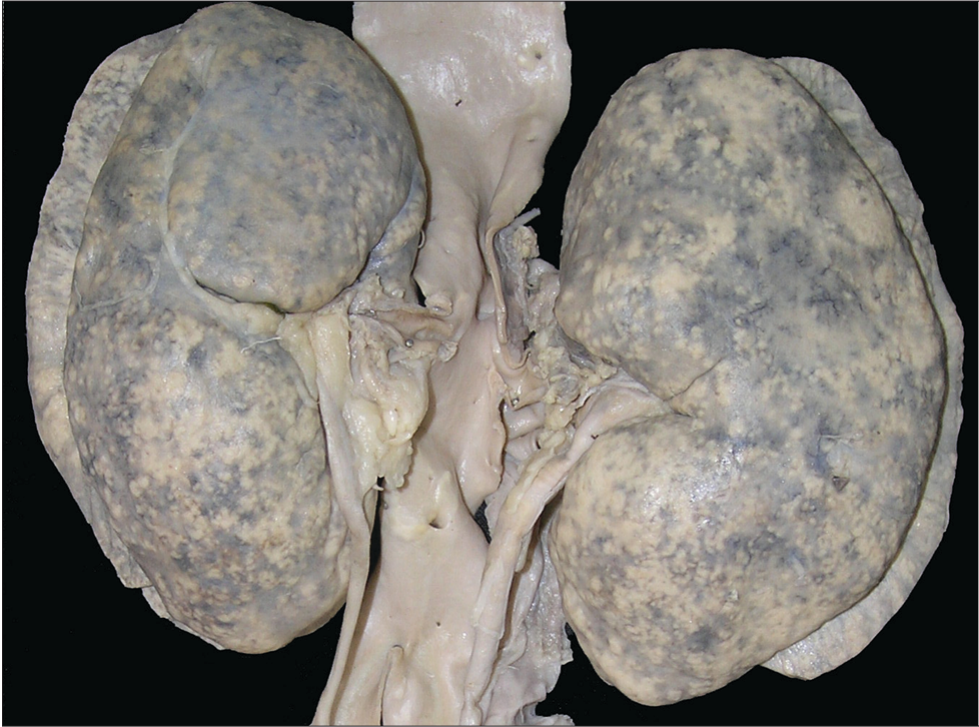
The surface of both kidneys showed alternate hemorrhagic and pale areas

**Fig. 4:**
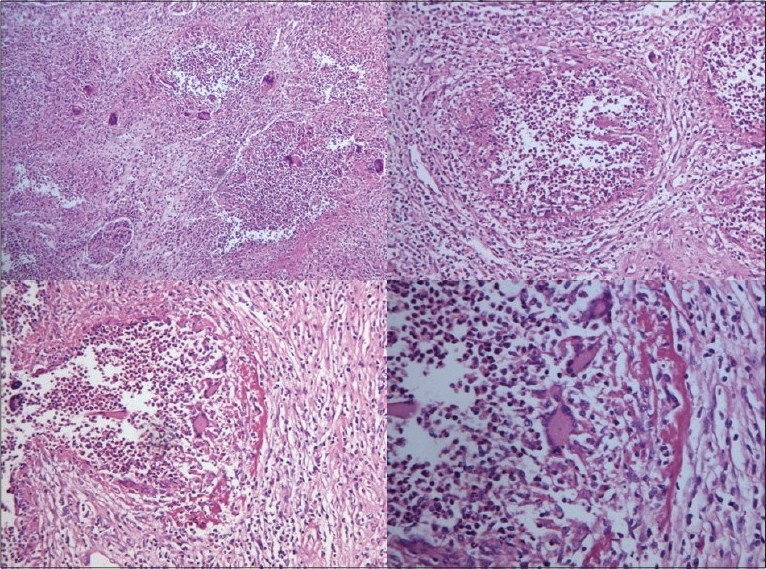
Microscopy of kidney showed numerous suppurative granulomata

**Fig. 5:**
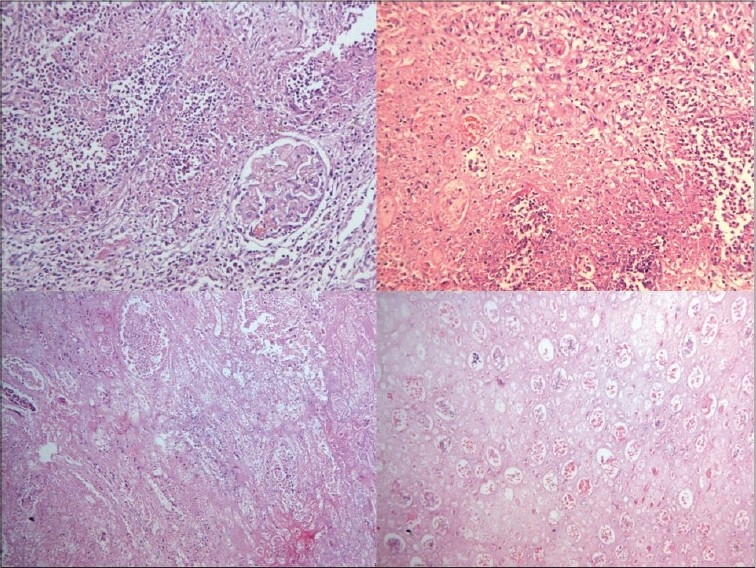
Bland infracted areas with many necrotic glomeruli

**Fig. 6:**
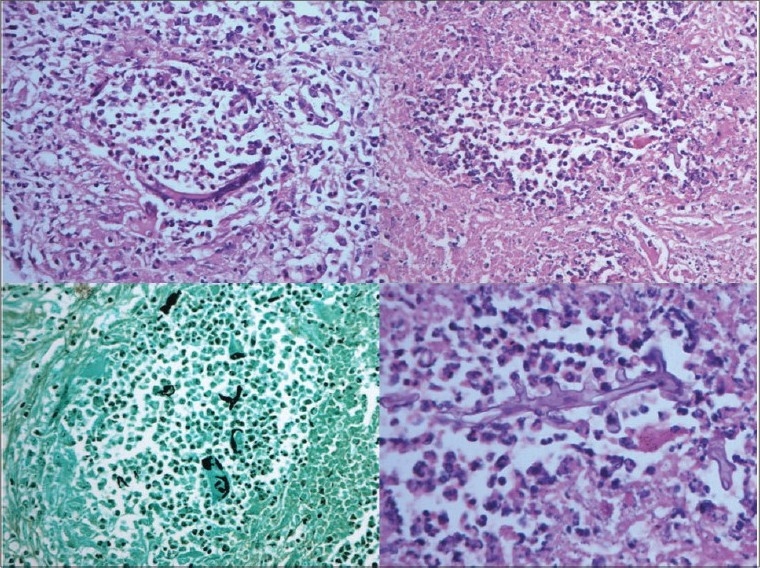
Many fungal profiles within the giant cells and broad aseptate hyphae of zygomycosis on Grocott stain

**Fig. 7:**
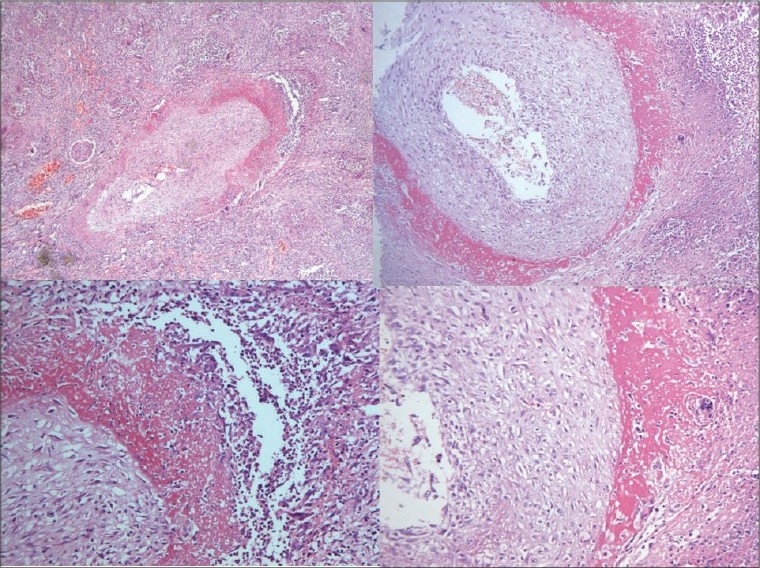
Evidence of vasculitis in the form of fibrinoid necrosis of blood vessels

**Kidneys** - Both kidneys were markedly enlarged and each measures 15 × 9 × 5 cm. The capsule can be stripped of easily. The surface showed alternate hemorrhagic and pale areas [Fig. 3]. Microscopy showed numerous suppurative granulomata [Fig. 4], with many bland areas including necrotic glomeruli [Fig. 5]. Many fungal profiles were seen within the giant cells [Fig. 6]. Special stains revealed broad aseptate hyphae confirming to the morphology of zygomycosis [Fig. 6]. Many vessels showed evidence of vasculitis [Fig. 7]. Splendore-Hopple phenomenon was noted.

**Fig. 8:**
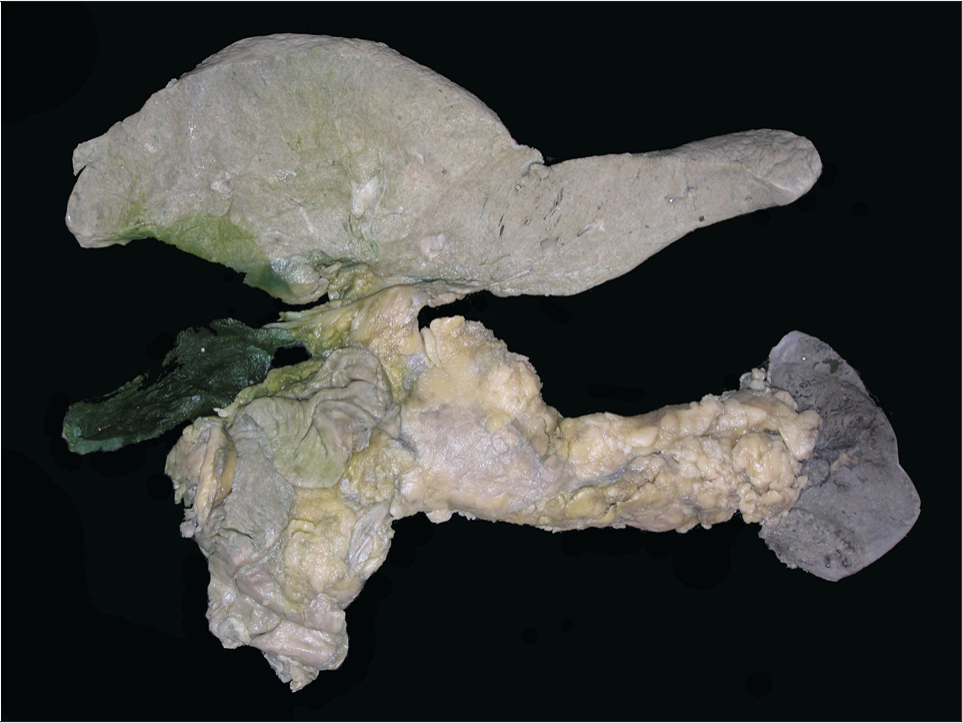
Composite specimen of liver, spleen, C-loop of duodenum and pancreas showing mild wrinkling of capsular surface of the liver

**Fig. 9:**
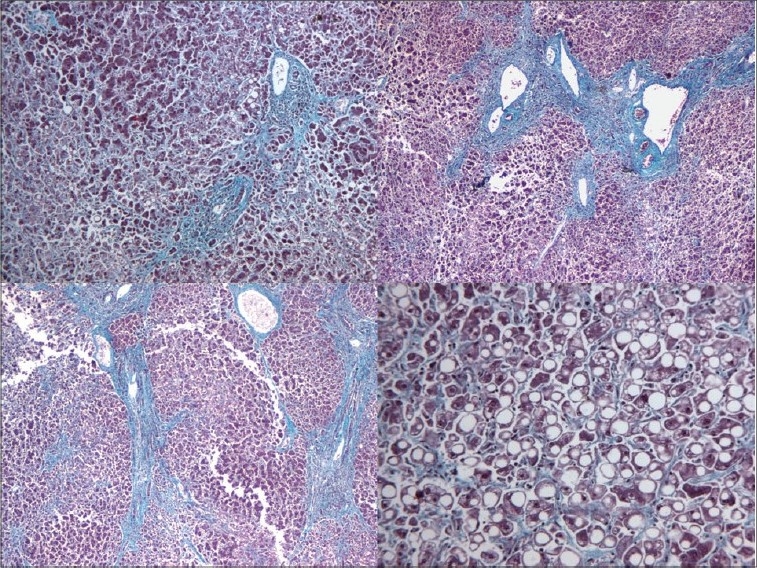
Extensive porto-portal and porto-central bridging with pericellular fibrosis and fatty change

**Fig. 10:**
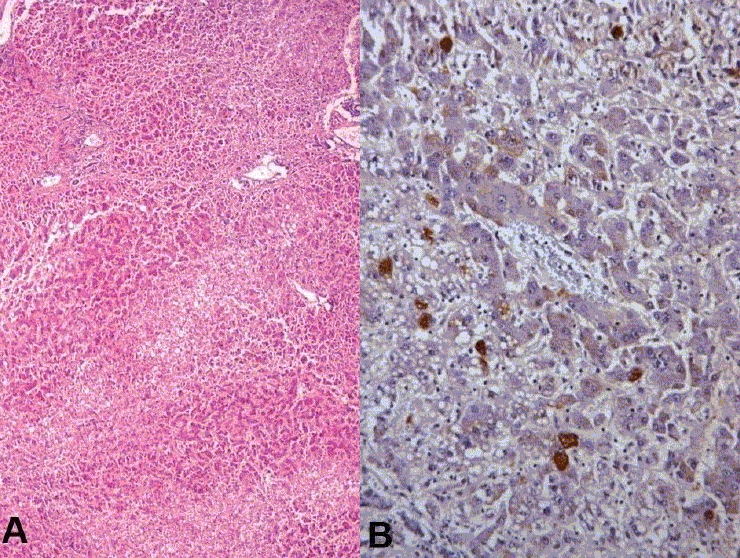
Fatty change with mild portal inflammation (A) and hepatitis B surface antigen positive hepatocytes on immunohistochemistry (B)

**Liver (850 g)** - The capsular aspect showed mild wrinkling [Fig. 8]. The portal tracts were irregularly fibrotic with porto-portal and porto-central bridging fibrosis [Fig. 9]. Fatty change was noted with patchy pericellular fibrosis [Fig. 9]. The hepatic parenchyma showed centrizonal fatty change with mild portal inflammation [Fig. 10A]. Immunostaining for hepatitis B surface antigen was positive in some hepatocytes [Fig. 10B]. The features were those of chronic hepatitis, hepatitis B related with Stage 4 fibrosis.

**Fig. 11:**
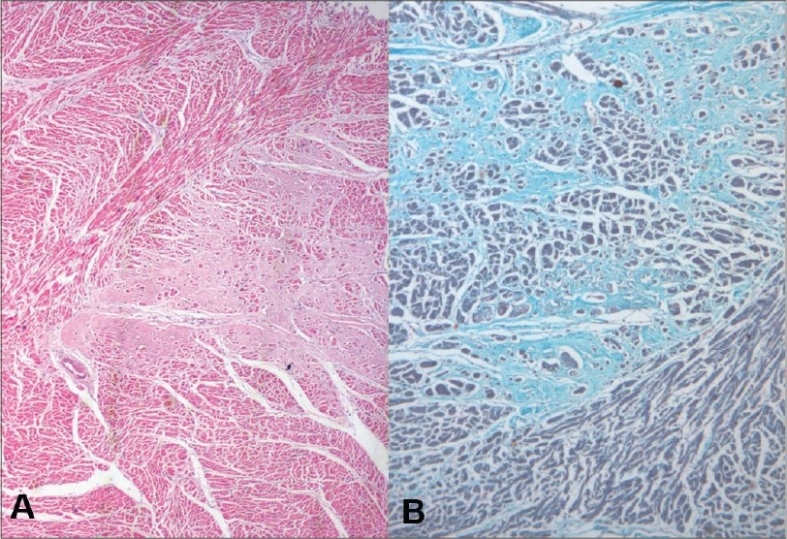
Myocardial fiber hypertrophy and interstitial fibrosis on H and E (A) and on Masson's trichrome (B)

**Heart (370 g)** - Left ventricular hypertrophy was noted. The left anterior descending artery showed 50-70% occlusion. The myocardial fibers showed hypertrophy with focal interstitial fibrosis [Fig. 11A,B].

**Fig. 12:**
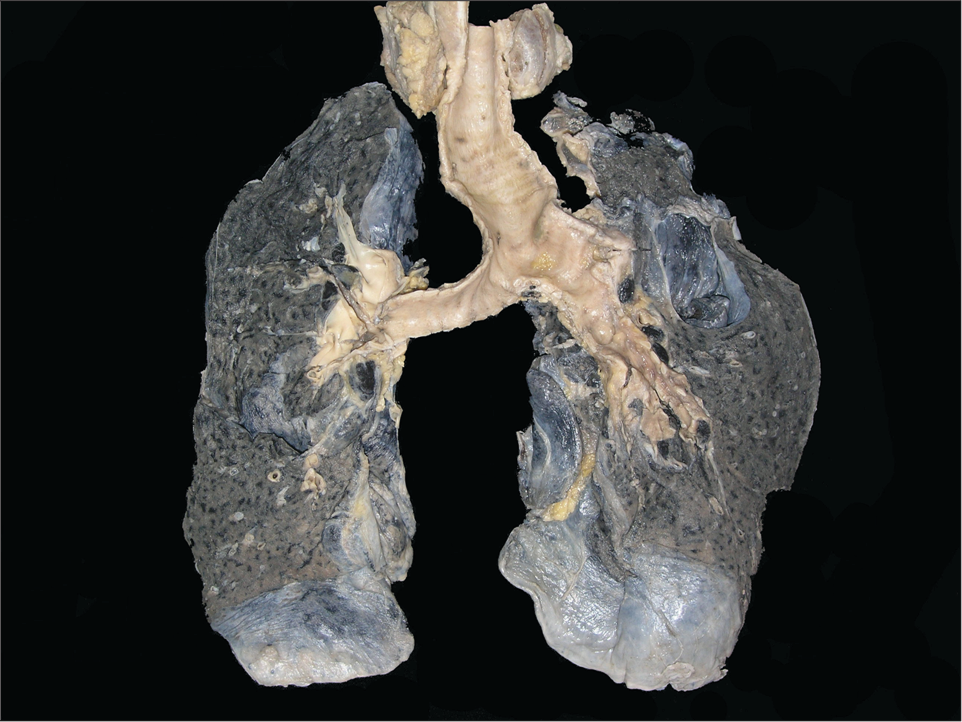
Cut surface of both lungs showed decortication of the upper lobe of right lung

**Fig. 13:**
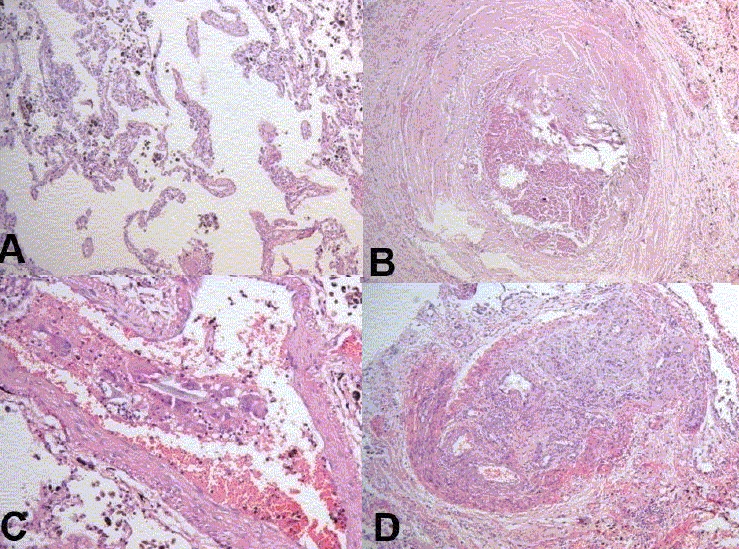
Microscopy showed emphysematous changes (A), evidence of old healed tuberculosis (B), foreign body embolus (C) and complex organized thrombus in one artery (D)

**Fig. 14:**
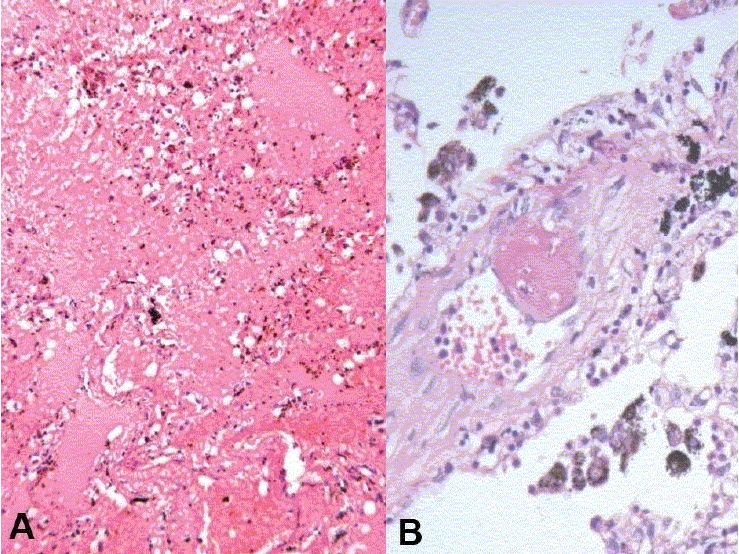
Microscopy showed evidence of pulmonary hemorrhage (A) and thromboembolism (B)

**Lungs (650 g)** - These were hyper inflated. Right upper lobe was decorticated while removal [Fig. 12]. A few calcified areas measuring 0.3-0.5 cm were also noted. Microscopically, the lung showed emphysematous changes [Fig. 13A], evidence of old-healed tuberculosis [Fig. 13B]. One artery also showed foreign body embolus [Fig. 13C]. Complex organized thrombus was also seen in one artery [Fig. 13D]. It also showed hemorrhagic areas [Fig. 14A] and evidence of pulmonary thromboemboli [Fig. 14B].

**Spleen (200 g)** - Red pulp congestion was noted.

**Brain** - No abnormality detected

**GI Tract** - Incidental Meckel's diverticulum noted

## Final Autopsy Diagnosis

1. Isolated bilateral renal zygomycosis
2. Old healed pulmonary tuberculosis, pulmonary emphysema, pulmonary thromboemboli with areas of pulmonary hemorrhage
3. Chronic hepatitis, hepatitis B related with Stage 4 fibrosis
4. Left ventricular hypertrophy

*Dr. Arunaloke Chakraborti, Professor of Medical Microbiology :* My concern here is little different type of tissue reaction than usual, where we see acute neutrophilic infiltrate or infarct. Now in the last 3-4 years, even the case reports have come in the literature that show chronic granulomatous reaction seen in cases of zygomycosis. It will be interesting to know whether it is because of host factor or it is difference in type of organism. Apophycomycosis is known to produce more chronic granulomatous reaction. Although in this particular case we were not able to isolate fungus, it is very interesting observation to know the tissue reaction and whether it is the host factor or the organism, which plays an important role. Chronic hepatitis could be one of the important predisposing factors as described in aspergillosis. Earlier people have not looked into these aspects and have not thought that hepatitis and cirrhosis can be a predisposing factor for systemic disseminated fungal disease.

*Dr. K Joshi, Professor and Head of Histopathology:* Actually when Dr. Naveen Kalra showed CT, I thought, despite not being a clinician that when in bilaterally enlarged kidneys, you see an extension into the perinephric areas, then isolated renal zygomycosis becomes a differential diagnosis, as we have seen in our previous cases. Most of the other cases have been very acute and had large extension into the perinephric fat, acute vascular involvement, and no reaction or very minimal neutrophilic reaction in the renal parenchyma. But this is the third case where we have very mild vascular reaction, mild extension to perinephric fat, and very dominant chronic granulomatus reaction. There are two pathologies, which we are seeing in isolated renal zygomycosis.

*Dr. Sakhuja:* I think if a CECT would have been done during life, it should have been easy to pick up the diagnosis. I don't know why a non-contrast CT scan was preferred by the treating unit. Probably, they were misled by the finding of mild hydronephrosis on the ultrasound and that is why they were looking for an etiology for hydronephrosis and went ahead with per-cutaneous nephrostomy as well.

*Dr. Chugh:* I think Dr. Chakraborti and Dr. Joshi very rightly highlighted the striking feature of this case. In that way they used to show us very large number of hyphae in every patient and here there is predominant chronic granulomatous reaction in enlarged kidneys. There is hardly any infarct like picture which you see and there are only a few hyphae.

*Dr. Das:* There were lots of hyphae in this case as well.

*Dr. Chugh:* Still, I believe there has to be something very peculiar and this disease has gone on for some time, with the kind of granulomatous reaction what we are seeing. Therefore one needs to look, what is happening in the body as far as mechanisms are concerned in this disease. We yet know so little about this disease and we need to know more about this as it is a very peculiar case and it looks much different from what we have seen and then why only confined to the kidneys. It is an infection, which involves the blood vessels and yet we are not seeing any lesion in the lungs, in the liver or anywhere. We have not seen the brain of course. Why it is confined to the kidneys, there must be again some reasons of which we are totally ignorant today.

*Dr. Joshi:* One of the pictures that Dr. Das has shown had infarction in renal papilla. There is papillary necrosis in this kidney and within the tubules of papilla I could see live fungal hyphae as we have seen in other cases of isolated renal zygomycosis. So I think, where there is infarction or tissue necrosis, the zygomycosis overgrow in the tissue. But this is a subacute/chronic granulomatous type of renal zygomycosis, but in areas of infarction the number of fungi would probably be much more than seen in a granulomatous reaction.

*Dr. Kalra:* As has already been highlighted, CECT would have shown us either presence of abscesses or bilateral enlarged non-functional kidneys as we have seen in previous cases of renal zygomycosis. There was actually apprehension of renal dysfunction, so contrast was held back. Probably even in such cases we can do contrast enhanced MRI. At present there are reports that CE-MRI would not be absolutely safe for the kidneys, but still that can be resorted to in some problematic cases to resolve the issue.

*Dr. Kim Vaiphei, Additional Professor of Histopathology:* My comment is with regard to granulomatous reaction to the fungus. Quiet a few years back there was a young male who had presented with generalized lymphadenopathy and thought to be lymphoma and biopsy had shown aspergillus with extensive granulomatous response. We were writing up the case as a case report and during that time, we happened to go though the literature in details about the granulomatous response to fungus in lymph node in immunocompetent host. Granulomatous response actually occurs when the fungus is a ghost or is a dead fungus and granulomatous response will not be seen in a viable fungus. So when fungus is dead or a dying fungus, granulomatous response will be seen and that too in an immunocompetent host, only then host will be able to kill the fungus and will have granulomatous response, not otherwise. This is with respect to aspergillus, possibly the same theory will be true in respect to mucormycosis though we have been seeing lots of cases in immunecompromised condition, and have been seeing different tissue responses.

*Dr. Sakhuja:* As regards the use of contrast is concerned in this patient, I won't hesitate to use the contrast at all as the patient was already anuric, so I don't see what nephrotoxicity we were apprehensive about and it is important to reach a diagnosis. So I think, by avoiding contrast, you are failing to reach a diagnosis as a consequence of that.

*Dr. Das:* In our last nine cases, there was no predisposing condition, even though they behaved in a different way. I don't know the basic reason for this. In this case, we don't have trace of any fungus in any other organ. Another thing is the host response, there is a lot of reaction surrounding these granulomas, the fibrinoid areas, you see there are so many types of granulomatous responses, also you can see so many vessels are involved and have fibrinoid necrosis of vessel wall, so this is a true infection (zygomycosis) related vasculitis. Several hyphae were present in center of all suppurative granulomas. Zygomycetes is a large fungus and you don't see it like *Candida*. But what is really striking that in adults it is always the zygomycetes which is involving the kidney when the isolated renal involvement is there. When it is part of generalized involvement, kidney is involved by any fungus.

*Dr. Aggarwal:* Pathogenesis of a granuloma is always the host response to a persistent antigen either dead or alive, so there is no question, that it is a fungus-related granuloma. Fungus has elicited a granulomatous response from the host. So we cannot say that fungus has caused the granulomas, the granulomas are always caused by the host because of persistent fungal infection, which can be dead or alive.

*Dr. Joshi:* I think when it is a question of isolated renal zygomycosis, PGI actually gets the credit for defining this disease. Our experience is published in largest case series from this institute.

*Dr. Sakhuja:* The question that has already been raised, why only the kidneys, is it just because of high renal blood flow.

*Dr. Jha, Additional Professor of Nephrology:* We have indeed seen large number of cases of isolated renal zygomycosis, but this phenomenon is not limited to PGI and has been reported from elsewhere in the world. In the *Journal of American Society of Nephrology*, a series of about 30 cases was published more than 10 years back, not from one institute of course, but collected from reports of cases from multiple centers around the world. Now, the reason why mucor specifically localizes to the kidneys is an enigmatic question to which still there is no answer. This is a fungus, which might get attracted to preexisting injuries, is it possible that there is minor injury or infection and somehow there is a lot of fungus which homes into this area. It is something which is possible, but there is no way to prove or disprove it unless some one does some kind of experimental work and shows that this happens.

*Dr. A Rajwanshi, Professor & Head of Cytology:* Nowadays we are also seeing large number of cases of mucormycosis referred to us from emergency for performing a FNA and FNA does help in making a diagnosis in those cases. It requires a high degree of suspicion, that we are suspecting mycormycosis. All this can be diagnosed on FNA even in morbid patient, where biopsy cannot be done.

*Dr. Chakraborti:* The western literature has not accepted isolated renal zygomycosis as an entity quite clearly. We must emphasize this entity; in international meetings suggestions are made that we have not looked into other organs properly. We can try and see whether this is an ascending infection. We can try in some animal model. This is a very interesting entity and this has been reported from PGI because of autopsy, now a number of cases are diagnosed antemortem. I think this particular entity should be very clearly mentioned as a separate entity and should be recognized in that way.
